# Medical Court Martial

**Published:** 1847-01

**Authors:** 


*Medical Court Martial.*—A kind of medical court-martial has been lately held at the hospital of Val de Grace, under the presidency of Colonel François, to try a surgical pupil of the hospital on a charge of having caused the death of a patient by giving him an overdose of opium.

The deceased, a military patient, was admitted into the hospital for ophthalmia, and the physician prescribed for him a sudorific syrup; but it so happened, that the first dose which was given to him contained about *an ounce of the tincture of opium!* (thirty grammes.) The man swallowed it, and died in three quarters of an hour, violently convulsed. The minister of war ordered an investigation of the circumstances.

M. Fretin, the accused, was interrogated on the allegation of his having labelled a bottle, containing tincture of opium, "sudorific syrup," but he denied that he had made any mistake, and contended that he was not responsible for the result. The accused was defended by counsel, and the court, after hearing the defence, acquitted him of the charge.—*Journal de Medecine..*

*** The case is chiefly remarkable from the fact that it is perhaps the most rapid instance of poisoning by opium on record.—*London Med. Gaz.*

